# Endothelium-Dependent Vasorelaxant Effect of *Prunus Persica* Branch on Isolated Rat Thoracic Aorta

**DOI:** 10.3390/nu11081816

**Published:** 2019-08-06

**Authors:** Bumjung Kim, Kwang-Woo Kim, Somin Lee, Cheolmin Jo, Kyungjin Lee, Inhye Ham, Ho-Young Choi

**Affiliations:** 1 Department of Herbal Pharmacology, College of Korean Medicine, Kyung Hee University, Seoul 02447, Korea; 2 Department of Herbal Pharmacology, Graduate School, Kyung Hee University, Seoul 02447, Korea; 3 Department of Biomedical Science and Technology, Graduate School, Kyung Hee University, Seoul 02447, Korea

**Keywords:** *Prunus persica*, hypertension, vasorelaxation, potassium channel, angiotensin II

## Abstract

Peach (*Prunus persica* (L.) Batsch) is a popular fruit consumed by people worldwide, owing to its pleasant flavor and high mineral nutrient content. A few plants from the genus *Prunus*, such as *Prunus yedoensis*, *Prunus cerasus*, and *Prunus serotina* have shown vasorelaxant and vasodilatory effects, to date, no study has investigated the vasorelaxation effects of the *P. persica* branch extract (PPE). The vasorelaxant effect of PPE was endothelium-dependent, and it was related to the NO-sGC-cGMP, vascular prostacyclin, and muscarinic receptor transduction pathway. K^+^ channels, such as the BK_Ca_, K_V_, and K_ATP_ channels, were partially associated with PPE-induced vasorelaxation. PPE was effective in relaxing serotonin (5-HT)- or angiotensin II-induced contraction; furthermore, PPE attenuated Ca^2+^-induced vasoconstriction by IP_3_ receptors in the SR membrane, but its vasorelaxant effect was not associated with the influx of extracellular Ca^2+^ via receptor-operative Ca^2+^ channels or voltage-dependent Ca^2+^ channels. Recognizing the rising use of functional foods for hypertension treatment, our findings imply that PPE may be a natural antihypertensive agent.

## 1. Introduction

Hypertension, also known as high or raised blood pressure, is a major cardiovascular disease (CVD) presenting high global health risk [1]. CVD is a leading cause of death worldwide, with hypertension provoking an estimated 9.4 million deaths per year; furthermore, 560 million extra people are expected to be affected by hypertension between 2000 and 2025 [2]. Hypertension is associated with substantial mortality, which can be dramatically reduced by the long-term control of blood pressure [3]. However, despite high levels of awareness and appropriate antihypertensive pharmacotherapy, hypertension control rates are still low [4].

Patients with hypertension can be treated successfully using various antihypertensive drugs as monotherapy or in combination therapy. Despite their efficacy, these medications can exhibit minor or even severe side effects: Beta-blockers may worsen symptoms of asthma and other lung diseases; diuretics can provoke reversible impotence and gout attacks; angiotensin-converting enzyme (ACE) inhibitors can induce persistent dry cough and angioedema; calcium channel blockers (CCB) can cause peripheral edema, headache, flushing, and tachycardia [5].

Numerous medicinal plants or herbal medicines have been used throughout the history of mankind for the treatment of various diseases. During the last few decades, public interest in medicinal plants has increased, because of their favorable side effect profile, low cost, and the emerging scientific evidence for their clinical utility [6]. Furthermore, the global use of herbal medicines with various pharmacological activities for CVD management and treatment is expanding [7].

*Prunus persica* (L.) Batsch, belonging to the Rosaceae family, is a deciduous tree native to the region of Northwest China, which is currently widely cultivated. Peach is a popular fruit consumed by people worldwide due to its pleasant flavor and high mineral nutrient content [8].

The *P. persica* seed is well known as a traditional medicine (Persicae Semen; Do-in in Korean, Taoren in Chinese) in Korea, China, Japan, and other Asian countries. It has traditionally been used for promoting blood flow, dispelling blood stasis, moistening the intestines, and relieving constipation [9]. Various chemical compounds are contained in this herbal medicine including amygdalin, cyanogenic glycosides, prunasin, emulsion, glycerides, and sterols [10]. Furthermore, several phenolic compounds have been isolated from *P. persica* leaves, such as caffeic acid, chlorogenic acid, kaempferol, *p*-coumaric acid, prussic acid, quercetin, quercitrin, quinic acid, tannin, and ursolic acid [11].

Previous pharmacological studies have demonstrated that *P. persica* exhibits an anti-tumor promoting [10], acetylcholinesterase inhibitory [12], anti-allergic inflammatory [13], anti-oxidant [14], and anti-hepatocellular carcinoma activity [15]. A few plants from the genus *Prunus*, such as *P. yedoensis* [16], *P. cerasus* [17], and *P. serotina* [18], have shown vasorelaxant and vasodilatory effects. Although a cardiovascular protective activity of peach by inhibiting angiotensin II-induced signal transduction has been previously reported [19], to date, no study has investigated the vasorelaxation effects of the *P. persica* branch extract (PPE).

## 2. Materials and Methods

### 2.1. Chemicals and Reagents

Modified Krebs-Henseleit (KH) buffer powder, phenylephrine (PE), potassium chloride (KCl), acetylcholine (ACh), *N*_ω_-Nitro-L-arginine methyl ester hydrochloride (L-NAME), 1*H*-[1,2,4]Oxadiazolo [4,3-a]quinoxalin-1-one (ODQ), methylene blue (MB), indomethacin, atropine, tetraethylammonium (TEA), 4-aminopyridine (4-AP), glibenclamide, serotonin hydrochloride (5-HT), angiotensin II (Ang II), calcium chloride (CaCl_2_), ethylene glycol-bis(2-aminoethylether)-*N*,*N*,*N*′,*N*′-tetraacetic acid (EGTA), and dimethyl sulfoxide (DMSO) were purchased from Sigma Aldrich (St. Louis, MO, USA). All other reagents were of analytical purity.

### 2.2. Plant Material and Extraction

*P. persica* was collected at Eumseong-gun, Chungcheongbuk-do, Republic of Korea, in March 2018. This plant was identified by Professor Kyungjin Lee of Kyung Hee University. Voucher specimens of the collected plants were deposited in the herbarium of the College of Korean Medicine, Kyung Hee University, Seoul, Republic of Korea. Dried branches (300.0 g) of *P. persica* were extracted two times with 3 L 70% ethanol (EtOH) for 3 h in a reflux apparatus at 70 ± 5 °C. After reflux and filtration, the extract was evaporated using a rotary vacuum evaporator at 60 °C and lyophilized by using a freeze-dryer to yield a dark brown residue (50.0 g) of crude extract. The dried sample of PPE was accurately weighed (0.1 g), dissolved in 1 mL KH buffer, and then placed in an ultrasonic bath for 10 min to break apart any remaining particulate matter.

### 2.3. Animals and Preparation of Rat Aortic Rings

Sprague-Dawley rats (male, 240 g–260 g, eight weeks old) were purchased from Raonbio (Yongin, Gyeonggi Province, Korea) and reared under standard laboratory conditions (temperature: 22 ± 2 °C, light: 07:00–19:00) and were given food and water *ad libitum*. All the animal studies followed the animal welfare guidelines and were approved (KHUASP (SE)-18-074) by the Kyung Hee University Institutional Animal Care and Use Committee.

Male SD rats were anesthetized by ether inhalation, and their thoracic aortae were isolated and placed in a KH buffer [composition (mM): NaCl, 118.0; KCl, 4.7; MgSO_4_, 1.2; KH_2_PO_4_, 1.2; CaCl_2_, 2.5; NaHCO_3_, 25.0; and glucose, 11.1; pH 7.4] by bubbling with a gas mixture of 95% O_2_—5% CO_2_ at 37 °C. The aortae were removed free of connective tissue and fat, and cut into approximately 2 mm long, and then suspended in organ chambers containing 10 mL KH buffer at 37 °C. Each arterial ring was suspended between two stainless steel hooks connected to an isometric force transducer to measure the tension. After incubation under no tension for 30 min, the aortic segments were equilibrated for 40 min at a resting tension of 1.2 g with changes of fresh buffer every 15–20 min. Changes in isometric tension of aortic rings were obtained via isometric transducers connected to a Powerlab Data Acquisition System with the Lab Chart software version 8.0 (Sydney, Australia). When required, the endothelium was removed by gentle rubbing of the lumen with a thin cotton swab. Integrity of the endothelium was confirmed when ACh (10 μM) caused greater than 70% relaxation after pre-contraction by PE (1 μM). Ca^2+^ free extracellular solutions were prepared by omitting CaCl_2_ and adding EGTA (1 mM). We applied the same concentration and equilibration time for the experimental period.

### 2.4. Experimental Protocols

#### 2.4.1. Effect of PPE on Endothelium-Intact and Endothelium-Denuded Aortic Rings Pre-Contracted by PE

PPE (0.5, 1, 2, 5, and 10 μg/mL) activity on endothelium-intact and endothelium-denuded aortic rings pre-contracted by PE (1 μM) was determined. The relaxant effect of PPE was calculated as a percentage of the relaxation in response to PE on the aortic rings.

#### 2.4.2. Effect of PPE on Endothelium-Intact Aortic Rings Pre-Incubated with L-NAME, ODQ, MB, Indomethacin, Atropine, Various Potassium Channel Blockers

The PPE (0.5–10 μg/mL) effect on the nitric oxide (NO) synthesis pathway in endothelium-intact aortic rings pre-incubated with L-NAME (10 μM), ODQ (10 μM), MB (10 μM), indomethacin (1 μM), atropine (1 μM), TEA (5 mM), 4-AP (1 mM), or glibenclamide (10 μM) for 20 min before PE (1 μM) pre-contraction was studied. Compared to the control (not treated with drugs), the relaxant effect of PPE was calculated as a percentage of the relaxation in response to the drugs pre-treatment on the aortic rings.

#### 2.4.3. Effect of PPE on Endothelium-Intact Aortic Rings Pre-Contracted by 5-HT

The relaxant effect of PPE (0.5–10 μg/mL) on endothelium-intact aortic rings pre-contracted by 5-HT (10 μM) was investigated. The relaxant effect of PPE was calculated as a percentage of the relaxation in response to 5-HT on the aortic rings.

#### 2.4.4. Effect of PPE Pre-Treatment on Ang II-Induced Contraction

The inhibitory role of PPE (10 μg/mL) on endothelium-intact aortic rings contracted by adding cumulative concentrations of Ang II (10^−9^–10^−6^ M) was analyzed. After aortic rings were pre-incubated for 20 min in the absence (control) or presence of PPE, contraction of the aortic rings was evoked by graded Ang II concentrations, and their responses was calculated as a percentage of the response of PPE to the contraction induced by Ang II.

#### 2.4.5. Effect of PPE on Extracellular Ca^2+^-Induced Contraction

The PPE (10 and 20 μg/mL) inhibitory activity on extracellular Ca^2+^-induced contractions through receptor-operative Ca^2+^ channels (ROCCs) or voltage-dependent Ca^2+^ channels (VDCCs) by PE or KCl pre-treatment, respectively, was investigated. We examined the contraction response by the influx of extracellular CaCl_2_ (0.3–10 mM) on endothelium-denuded aortic rings by PE (1 μM) or KCl (60 mM) pre-treatment in Ca^2+^-free KH buffer without (control) and with PPE pre-incubation for 10 min. Compared to the control (not treated with PPE), the contraction responses induced by CaCl_2_ were calculated as a percentage in the absence (control) and presence of PPE pre-treatment.

#### 2.4.6. Effect of PPE on Intracellular Ca^2+^ Release

The effect of PPE (10 and 20 μg/mL) on the intracellular Ca^2+^ release from the sarcoplasmic reticulum (SR) via the specific inositol triphosphate receptor (IP_3_R) was determined. We examined the contraction response by PE (1 μM) on endothelium-denuded aortic rings in the Ca^2+^-free KH buffer without (control) and with PPE pre-incubation for 10 min. Compared to the control (not treated with PPE), the contraction responses induced by PE were calculated as a percentage in the absence (control) and presence of the PPE pre-treatment.

### 2.5. Equation for Percentage Changes of Vasorelaxation

The equation for percentage changes of vasorelaxation after treatment with extracts on aortic rings were pre-contracted by PE or KCl as follows:

*Percentage of vasorelaxation* = [{(*A* − *C*) − (*B* − *C*)}/(*A* − *C*)] × *100* (1)

where *A* is the maximal contraction of aortic rings after pre-contraction by PE; *B* is the contraction of aortic rings with drug treatment; *C* is the contraction of aortic rings before pre-contraction by PE.

### 2.6. Data Analysis

Values of the outcome data are expressed as the mean ± standard error of mean (SEM). Statistical comparisons between experimental groups were made using the Student’s *t*-test with *p* < 0.05 accepted as being statistically significant. All statistical analyses were performed by using the SPSS statistical analysis software (version 23.0; SPSS Inc., Chicago, IL, USA).

## 3. Results

### 3.1. Vasorelaxant Effect of PPE on Endothelium-Intact and Endothelium-Denuded Aortic Rings Pre-Contracted by PE or KCl

**Figure 1:**
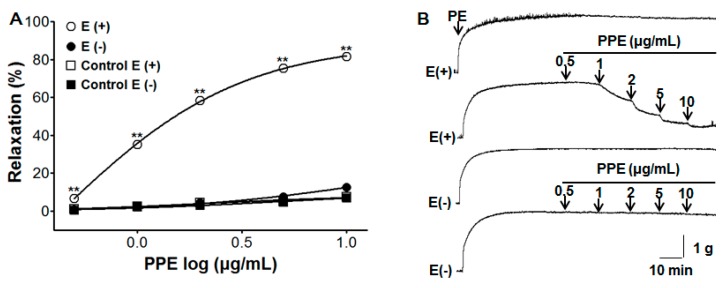
(**A**) Concentration response vasorelaxant effect for the *P. persica* branch extract (PPE) (0.5–10 μg/mL) on phenylephrine (PE, 1 μM) pre-contracted aortic rings with (Endo+) or without (Endo-) endothelium; (**B**) representative traces of vasorelaxant effect induced by PPE on aortic rings pre-contraction by PE. Values are expressed as mean ± SEM (*n* = 6–9). ** *p* < 0.01 compared to the control.

In this study, we found the optimal concentration of PPE to relax blood vessels by evaluating the results of several experiments. PPE caused concentration-dependent vasorelaxation on endothelium-intact but did not cause vasorelaxation on endothelium-denuded aortic rings pre-contracted by PE (1 μM). The maximal vasorelaxant effect in PE-induced contraction was 81.6 ± 0.4% and 12.6 ± 0.9% for endothelium-intact and endothelium-denuded aortic rings at the concentration of 10 μg/mL, respectively (Figure 1).

### 3.2. Vasorelaxant Effect of PPE on Endothelium-Intact Aortic Rings Pre-Incubated with L-NAME

**Figure 2:**
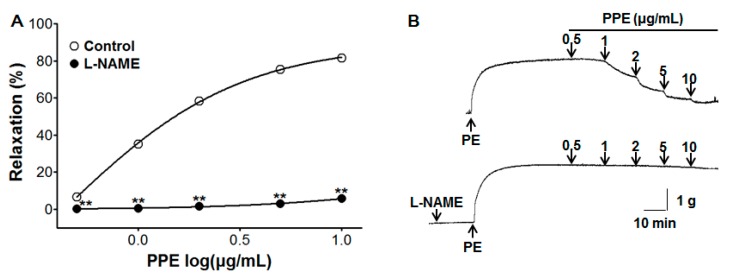
Concentration response (**A**) and representative traces (**B**) of vasorelaxant effect for PPE (0.5–10 μg/mL) in the absence (control) or presence of *N*_ω_-Nitro-L-arginine methyl ester hydrochloride (L-NAME, 10 μM) on phenylephrine (PE, 1 μM) pre-contracted aortic rings. Values are expressed as mean ± SEM (*n* = 5–9). ** *p* < 0.01 compared to the control.

Pre-incubation with L-NAME (10 μM) significantly inhibited PPE-induced vasorelaxation on endothelium-intact aortic rings pre-contracted by PE (1 μM). In the absence and presence of L-NAME, the maximal vasorelaxant effect was 81.6 ± 0.4% and 5.7 ± 0.6% at the concentration of 10 μg/mL, respectively (Figure 2).

### 3.3. Vasorelaxant Effect of PPE on Endothelium-Intact Aortic Rings Pre-Incubated with ODQ or MB

**Figure 3:**
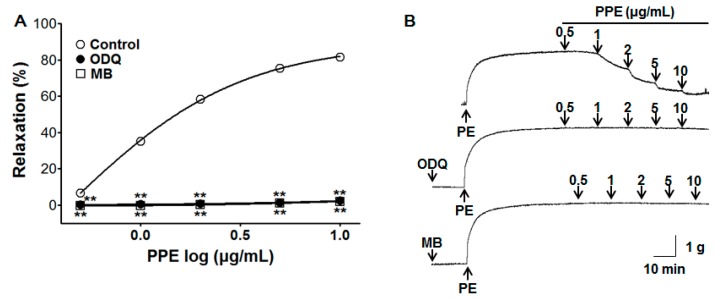
Concentration response (**A**) and representative traces (**B**) of vasorelaxant effect for PPE (0.5–10 μg/mL) in the absence (control) or presence of 1*H*-[1,2,4]Oxadiazolo [4,3-a]quinoxalin-1-one (ODQ, 10 μM) or methylene blue (MB, 10 μM) on phenylephrine (PE, 1 μM) pre-contracted aortic rings. Values are expressed as mean ± SEM (*n* = 6–9). ** *p* < 0.01 compared to the control.

To test the relaxant properties of PPE (0.5–10 μg/mL) in the cyclic guanosine monophosphate (cGMP) pathway, pre-incubation with ODQ (10 μM) and MB (10 μM) significantly inhibited PPE-induced vasorelaxation on endothelium-intact aortic rings pre-contracted by PE (1 μM). In the presence of ODQ and MB, the maximal vasorelaxant effect was 2.4 ± 0.3% and 2.0 ± 0.2% at the concentration of 10 μg/mL, respectively (Figure 3).

### 3.4. Vasorelaxant Effect of PPE on Endothelium-Intact Aortic Rings Pre-Incubated with Indomethacin

**Figure 4:**
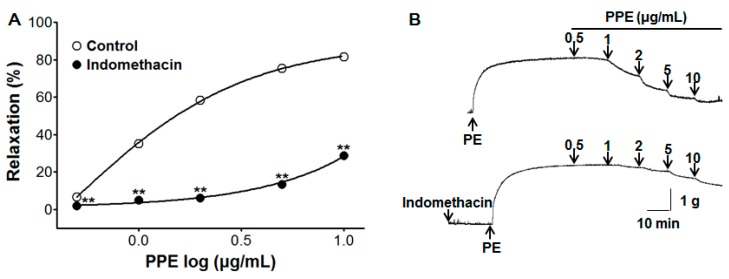
Concentration response (**A**) and representative traces (**B**) of vasorelaxant effect for PPE (0.5–10 μg/mL) in the absence (control) or presence of indomethacin (1 μM) on phenylephrine (PE, 1 μM) pre-contracted aortic rings. Values are expressed as mean ± SEM (*n* = 7–9). ** *p* < 0.01 compared to the control.

To test the activity of PPE (0.5–10 μg/mL) in the prostacyclin pathway, pre-incubation with indomethacin (1 μM) significantly inhibited PPE-induced vasorelaxation on endothelium-intact aortic rings pre-contracted by PE (1 μM). In the absence and presence of indomethacin, the maximal vasorelaxant effect was 81.6 ± 0.4% and 28.8 ± 1.9% at the concentration of 10 μg/mL, respectively (Figure 4).

### 3.5. Vasorelaxant Effect of PPE on Endothelium-Intact Aortic Rings Pre-Incubated with Atropine

**Figure 5:**
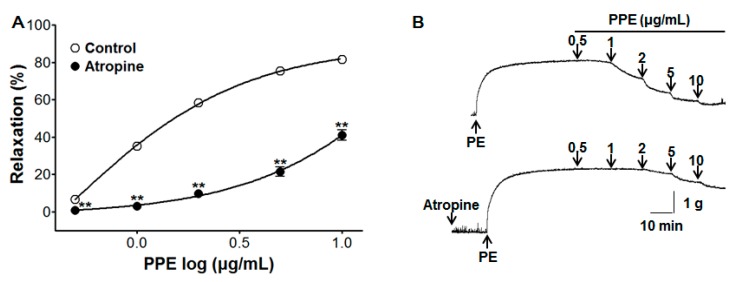
Concentration response (**A**) and representative traces (**B**) of vasorelaxant effect for PPE (0.5–10 μg/mL) in the absence (control) or presence of atropine (1 μM) on phenylephrine (PE, 1 μM) pre-contracted aortic rings. Values are expressed as mean ± SEM (*n* = 7–9). ** *p* < 0.01 compared to the control.

To test the relaxant effect of PPE (0.5–10 μg/mL) from the stimulation of muscarinic receptors, pre-incubation with atropine (1 μM) significantly inhibited PPE-induced vasorelaxation on endothelium-intact aortic rings pre-contracted by PE (1 μM). In the absence and presence of atropine, the maximal vasorelaxant effect was 81.6 ± 0.4% and 41.1 ± 2.8% at the concentration of 10 μg/mL, respectively (Figure 5).

### 3.6. Vasorelaxant Effect of PPE on Endothelium-Intact Aortic Rings Pre-Incubated with Various Potassium Channel Blockers

**Figure 6:**
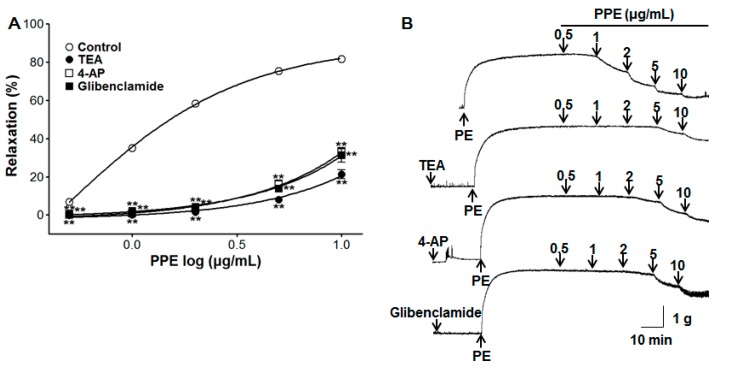
Concentration response (**A**) and representative traces (**B**) of vasorelaxant effect for PPE (0.5–10 μg/mL) in the absence (control) or presence of tetraethylammonium (TEA, 5 mM), 4-aminopyridine (4-AP, 1 mM), or glibenclamide (10 μM) on phenylephrine (PE, 1 μM) pre-contracted aortic rings. Values are expressed as mean ± SEM (*n* = 6–9). ** *p* < 0.01 compared to the control.

To test the role of PPE (0.5–10 μg/mL) in the potassium channel, pre-incubation with potassium channel blockers such as TEA (5 mM), 4-AP (1 mM), or glibenclamide (10 μM) significantly inhibited PPE-induced vasorelaxation on endothelium-intact aortic rings pre-contracted by PE (1 μM). In the presence of TEA, 4-AP, or glibenclamide, the maximal vasorelaxant effect was 21.2 ± 2.3%, 32.6 ± 2.1%, and 31.4 ± 3.9% at the concentration of 10 μg/mL, respectively (Figure 6).

### 3.7. Vasorelaxant Effect of PPE on 5-HT-Induced Contraction

**Figure 7:**
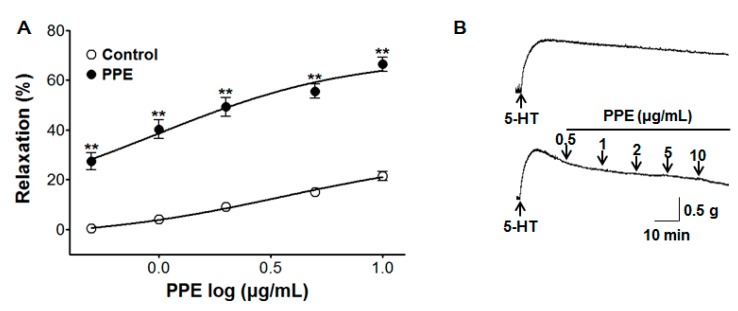
Concentration response (**A**) and representative traces (**B**) of vasorelaxant effect for PPE (0.5–10 μg/mL) in the absence (control) or presence of PPE on serotonin hydrochloride (5-HT, 10 μM) pre-contracted aortic rings. Values are expressed as mean ± SEM (*n* = 5–7). ** *p* < 0.01 compared to the control.

PPE caused concentration-dependent vasorelaxation on endothelium-intact aortic rings pre-contracted by 5-HT (10 μM). The maximal vasorelaxant effect was 66.4 ± 2.8% compared to the control group 21.5 ± 1.9% at the concentration of 10 μg/mL, respectively (Figure 7).

### 3.8. Inhibitory Effect of PPE Pre-Treatment on Ang II-Induced Contraction

**Figure 8:**
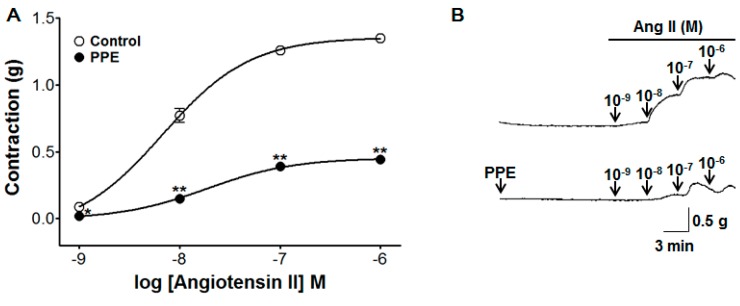
Inhibitory effect (**A**) and representative traces (**B**) of PPE (10 μg/mL) in the contraction induced by angiotensin II (Ang II, 10^−9^–10^−6^ M) on endothelium-intact aortic rings in the absence (control) or presence of PPE. Values are expressed as mean ± SEM (*n* = 7). * *p* < 0.05, ** *p* < 0.01 compared to the control.

An experiment was conducted to evaluate the inhibitory effect of PPE (10 μg/mL) on endothelium-intact aortic rings evoked by Ang II (10^−9^–10^−6^ M). PPE pre-treatment significantly attenuated the contraction induced by Ang II. The contraction was decreased to 0.44 ± 0.02 g compared to the control group 1.35 ± 0.03 g at Ang II 10^−6^ M concentration, respectively (Figure 8).

### 3.9. Inhibitory Effects of PPE on Extracellular Ca^2+^-Induced Contraction Through ROCCs or VDCCs

**Figure 9:**
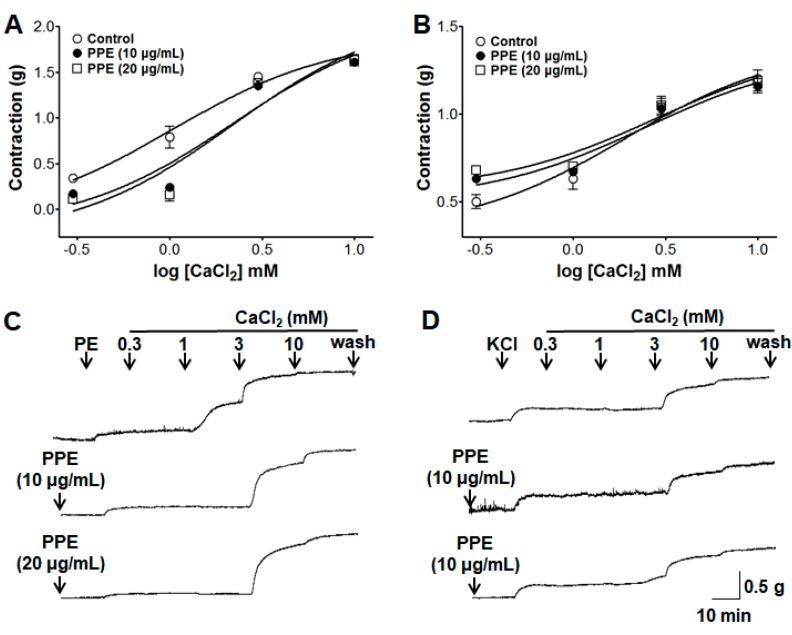
Inhibitory effect of PPE (10 and 20 μg/mL) in the contraction induced by extracellular CaCl_2_ (0.3–10 mM) on endothelium-denuded aortic rings that were pre-contracted with phenylephrine (PE, 1 μM) (**A**) or potassium chloride (KCl, 60 mM) (**B**) in the absence (control) or presence of PPE. (**C**,**D**) are representative traces under the indicated conditions. Values are expressed as mean ± SEM (*n* = 5–9).

The accumulative addition of CaCl_2_ (0.3–10 mM) induced the gradual increase of tension through ROCCs or VDCCs by PE (1 μM) or KCl (60 mM) pre-treatment on endothelium-denuded aortic rings in a Ca^2+^-free KH buffer. PPE (10 and 20 μg/mL) pre-incubation did not alter the contractions induced by extracellular CaCl_2_ (10 mM). The contraction at PPE (10 and 20 μg/mL) pre-incubation was 1.61 ± 0.04 g and 1.63 ± 0.06 g compared to the control group 1.65 ± 0.01 g on aortic rings pre-contracted by PE, respectively (Figure 9). The contraction at PPE (10 and 20 μg/mL) pre-incubation was 1.16 ± 0.04 g and 1.19 ± 0.06 g compared to the control group 1.20 ± 0.05 g on aortic rings pre-contracted by KCl, respectively (Figure 9).

### 3.10. Inhibitory Effects of PPE on Intracellular Ca^2+^ Release

**Figure 10:**
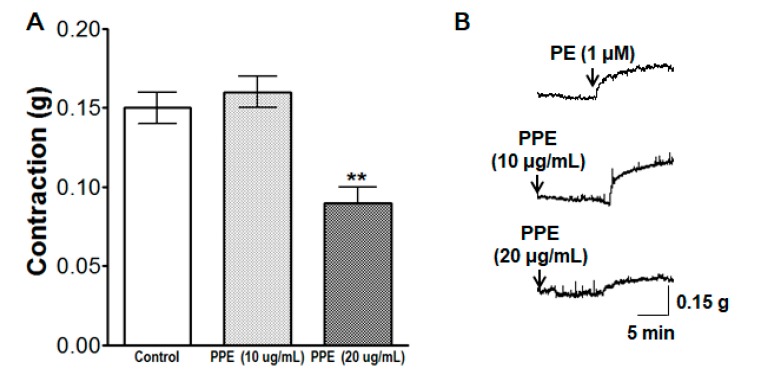
Inhibitory effect (**A**) and representative traces (**B**) of PPE (10 and 20 μg/mL) in the contraction induced by intracellular Ca^2+^ release from the sarcoplasmic reticulum (SR) via the specific inositol triphosphate receptor (IP_3_R) on endothelium-denuded aortic rings that were pre-contracted with phenylephrine (PE, 1 μM) in Ca^2+^-free KH buffer. Values are expressed as mean ± SEM (*n* = 6). ** *p* < 0.01 compared to the control.

PPE (10 and 20 μg/mL) pre-incubation for 10 min attenuated the contraction induced by PE (1 μM) on endothelium-denuded aortic rings in a Ca^2+^-free KH buffer. The contraction at PPE (20 μg/mL) pre-incubation was significantly decreased to 0.09 ± 0.01 g compared to the control group 0.15 ± 0.01 g (Figure 10).

## 4. Discussion

The *P. persica* (peach) fruit is widely consumed, and its seed is well known as a traditional medicine against blood stasis in Korea, China, and Japan [15]. Several medicinal plants belonging to the *Prunus* genus have shown vascular activities: *P. yedoensis* bark relaxed vascular blood vessels by activating the NO formation from L-arginine and the NO-cGMP pathway, and blocking Ca^2+^ entry through extracellular Ca^2+^ channels [16]; the *P. cerasus* fruit (Montmorency tart cherry) significantly lowered systolic blood pressure in men with early hypertension [17]; and *P. serotina* fruits (Black cherry) induced vasodilation by activating the NO/cGMP and H_2_S/K_ATP_ channel pathways [18]. The *P. persica* fruit (peach) has also shown cardiovascular protective effects by inhibiting angiotensin II-induced signal transduction in vascular smooth muscle cells (VSMCs) [19]. Here, we assessed for the first time the vascular activities of PPE by testing the vasorelaxant effect and investigating the associated mechanisms using an isolated organ-chamber technique. In the present study, the 70% ethanol PPE caused the concentration-dependent vasorelaxation of rat aortic rings pre-contracted by PE.

The endothelium plays a critical role in regulating the vascular function by secreting various endothelium-derived relaxing factors, such as NO and prostaglandins, endothelium-dependent hyperpolarization factors, or endothelium-derived contracting factors, including thromboxane and endothelin-1. The deficiency of these vasoactive agents, including relaxing factors, can cause the endothelial dysfunction. The endothelial dysfunction has been implicated in numerous pathological conditions, such as stroke, heart disease, vascular disease associated with vasoconstriction, inflammatory state, and thrombosis [20]. PPE exhibited a vasorelaxant effect on endothelium-intact aortic rings contracted by PE or KCl; however, the vasorelaxant activity of PPE on endothelium-denuded aortic rings was reduced. The data suggested that the vasorelaxant effect of PPE is endothelium-dependent.

NO, a gas synthesized endogenously from the amino acid L-arginine by NO synthase (NOS), causes vasodilation. In smooth muscle cells, NO activates soluble guanylate cyclase (sGC), which in turn increases cGMP, leading to the activation of cGMP-dependent protein kinases (PKG), an intracellular Ca^2+^ concentration reduction, and vasodilation induction [21]. To investigate endothelium-related vasorelaxation including the NO synthesis pathway and cGMP pathway, L-NAME (NOS inhibitor), ODQ, and MB (sGC inhibitor) were utilized. The vasorelaxant effects of PPE were reduced after pre-treatment with L-NAME, ODQ, or MB. These results imply an association of PPE vasorelaxant effects with the NO-sGC-cGMP pathway.

Prostacyclin (PGI_2_) and thromboxane are important vascular prostanoids, which are formed by cyclooxygenase from the arachidonic acid. PGI_2_ generates cyclic adenosine monophosphate (cAMP), leading to the induction of endothelial-dependent vasorelaxation [22]. Pre-incubation with indomethacin (a non-selective cyclooxygenase inhibitor) inhibited PPE-induced vasorelaxation, indicating that the PPE vasorelaxant effect is related to the vascular prostacyclin pathway.

Moreover, atropine, an antagonist of muscarinic acetylcholine receptors [23], affected the vasorelaxation induced by PPE, implying an association of the PPE vasorelaxant activity with the muscarinic receptor.

Vascular contraction and relaxation were also regulated by the membrane potential of arterial smooth muscle cells through K^+^ channels. The K^+^ channel opening causes hyperpolarization and relaxation of VSMCs, a subsequent blood flow increase, and a blood pressure decrease. There are four different K^+^ channel subtypes including Ca^2+^-activated K^+^ channels (BK_Ca_), voltage-gated K^+^ channels (K_V_), ATP-sensitive K^+^ channels (K_ATP_), and inward rectifiers K^+^ channels (K_IR_) [24]. To investigate potential K^+^ channel-related PPE-induced vasorelaxation, common K^+^ channel blockers such as TEA (BK_Ca_ blocker), 4-AP (K_V_ blocker), and glibenclamide (K_ATP_ blocker) were used. The vasorelaxant effect of PPE was partially attenuated by TEA, 4-AP, or glibenclamide pre-incubation. These results suggest that the vasorelaxant effects of PPE are partially related to K^+^ channels, such as BK_Ca_, K_V_, and K_ATP_ channels.

Serotonin, also known as 5-hydroxytryptamine (5-HT), is an autacoid synthesized primarily in the enterochromaffin cells of the intestine and in some areas of the brain. 5-HT is a key neurotransmitter and a vasoactive amine involved in the nervous, gastrointestinal, and cardiovascular systems [25]. 5-HT constricts the arteries by inhibiting the K_V_ and K_ATP_ channel activity in various vascular beds [26]. PPE caused concentration-dependent vasorelaxation of aortic rings pre-contracted by 5-HT, demonstrating that PPE is effective in relaxing the 5-HT-induced contraction.

The renin-angiotensin system (RAS) is a hormone system maintaining the effective circulating blood volume and blood pressure by regulating the total sodium content. Renin, a proteolytic enzyme synthesized and stored in the juxtaglomerular cells, splits a 10-amino acid fragment named angiotensin I (Ang I) from angiotensinogen. ACE, one of the key enzymes in the RAS, removes 2 amino acids from Ang I generating the octapeptide Ang II, which causes the vasoconstriction and aldosterone release [27]. Pre-treatment with PPE significantly attenuated the Ang II-induced contraction, implying that PPE may inhibit the Ang II–induced contraction of aortic rings with intact endothelium.

Both the Ca^2+^ release from intracellular Ca^2+^ stores and Ca^2+^ influx from the extracellular space through ROCCs or VDCCs in plasma membrane channels regulate the contraction and relaxation of the vascular smooth muscle [28]. PE, an α-adrenergic agonist, causes vascular contraction by releasing intracellular Ca^2+^ from the SR following activation of inositol 1,4,5-trisphosphate (IP_3_) receptors, as well as by entry of extracellular Ca^2+^ via ROCCs. However, KCl induces the vascular contraction as a result of extracellular Ca ^2+^ influx by the depolarization-induced opening of VDCCs [29]. PPE pre-incubation did not affect vasoconstriction induced by Ca^2+^ supplementation in the aortic rings pre-contracted with PE or KCl in Ca^2+^-free KH buffer. These results suggest that the vasorelaxant effects of PPE are not associated with the influx of extracellular Ca^2+^ via ROCCs or VDCCs. Moreover, PPE pre-incubation attenuated the contraction induced by PE on endothelium-denuded aortic rings in the Ca^2+^-free KH buffer, suggesting that PPE may inhibit Ca^2+^-induced vasoconstriction by IP_3_ receptors in the SR membrane. 

Recognizing the rising use of functional foods for hypertension treatment, our findings indicate that PPE may be a potential natural antihypertensive agent. However, since *P. persica* contains a variety of compounds, further in-depth studies are required for the isolation and identification of pharmacologically active molecules. 

## 5. Conclusions

In conclusion, (1) the vasorelaxant effect of PPE was endothelium dependent; (2) was related to the NO-sGC-cGMP, vascular prostacyclin, and muscarinic receptor transduction pathway; (3) K^+^ channels, such as the BK_Ca_, K_V_, and K_ATP_ channels, were partially related to the PPE-induced vasorelaxation; (4) PPE was effective in relaxing the contraction induced by 5-HT or Ang II; and (5) PPE attenuated Ca^2+^-induced vasoconstriction by IP_3_ receptors in the SR membrane; however, (6) the vasorelaxant effects of PPE were not associated with the influx of extracellular Ca^2+^ via ROCCs or VDCCs.
